# Efficacy of continuous plasma diafiltration therapy

**DOI:** 10.1186/cc13590

**Published:** 2014-03-17

**Authors:** T Komura, T Taniguchi, T Noda, M Okajima, S Kaneko

**Affiliations:** 1 Kanazawa University, Kanazawa, Japan

## Introduction

Acute liver failure (ALF) is a critical illness with high mortality. Plasma diafiltration (PDF) is a blood purification therapy in which simple plasma exchange is performed using a selective membrane plasma separator while the dialysate flows outside the hollow fibers. While several studies demonstrated that PDF therapy is a useful blood purification therapy for patients with ALF, PDF therapy is often difficult to employ in ALF patients complicated with multiple organ failure, especially in those with unstable hemodynamics. Furthermore, it is likely to re-occur immediately after PDF therapy. We developed continuous PDF (CPDF) as a new concept in PDF therapy, and assessed its efficacy and safety in ALF patients compared with conventional plasma exchange (PE) plus continuous hemodiafiltration (CHDF) therapy in this study.

## Methods

Ten ALF patients (gender: male/female = 6/4, age: 47 ± 14) employed CPDF therapy. The primary outcomes were altered liver function, measured by the model for end-stage liver disease (MELD) score, and total bilirubin and prothrombin time International Normalized Ratio (PT-INR), 5 days after CPDF therapy. Secondary outcomes included Sequential Organ Failure Assessment (SOFA) scores, 5 days after CPDF therapy, and the survival rate 14 days after this therapy.

## Results

The MELD score (34.5 to 28.0; *P *= 0.005), total bilirubin (10.9 to 7.25 mg/dl; *P *= 0.048), PT-INR (1.89 to 1.31; *P *= 0.084), and SOFA score (10.0 to 7.5; *P *< 0.039) were improved 5 days after CPDF therapy. Nine patients were alive and one patient died due to acute pancreatitis, complicated by ALF. The efficacy of CPDF therapy for maintaining liver function and renal function was not inferior to Pe plus CHDF therapy. Parameters of renal function such as the creatinine value were also improved 5 days after CPDF therapy. Circulation parameters such as mean arterial pressure and heart rate were maintained without inotropic and vasopressor support during the CPDF treatment period. The oxygenation index (PaO_2_/FiO_2_) as a measure of pulmonary function tended to increase after this treatment. We could employ this treatment without any adverse events, such as infections and unstable hemodynamics.

## Conclusion

In the present study, CPDF therapy safely supported liver function and generally improved the condition of critically ill patients with ALF.

